# Diagnostic Imaging of Primary Hepatic Neuroendocrine Tumors: A Case and Discussion of the Literature

**DOI:** 10.1155/2014/156491

**Published:** 2014-09-02

**Authors:** Trenton Kellock, Betty Tuong, Alison C. Harris, Eric Yoshida

**Affiliations:** ^1^Department of Medicine, University of British Columbia, 2775 Laurel Street, 11th Floor, Vancouver, BC, Canada V5Z 1M9; ^2^Department of Radiology, Vancouver General Hospital, 899 West 12th Avenue, Vancouver, BC, Canada V5Z 1M9; ^3^Department of Gastroenterology, Vancouver General Hospital, 899 West 12th Avenue, Vancouver, BC, Canada V5Z 1M9

## Abstract

Neuroendocrine tumors (NETs) are derived from neuroendocrine cells that are capable of producing functional peptide hormones. These tumors occur most frequently in the GI tract and lungs. GI NETs frequently metastasize into the liver, though NETs of primary hepatic origin are extremely rare. Ultrasound, CT, and MRI are typically all employed for characterization of these lesions but their appearance on diagnostic imaging can be highly variable. Reported here is an interesting case of a primary hepatic neuroendocrine tumor (PHNET), along with a discussion of the imaging characteristics of these tumors. Additionally, the current standards for definitive diagnosis and treatment of PHNETs are discussed.

## 1. Introduction

Neuroendocrine tumors (NETs) are tumors derived from neuroendocrine cells that are capable of producing functional peptide hormones. Typically, these tumors are found in the GI tract (55%) and lungs (30%), though they can also arise in the pancreas (2%), reproductive system (1%), biliary tract (1%), and head and neck (0.4%), among other areas [1]. Well-differentiated NETs were classically referred to as carcinoids; however, the WHO has reclassified these tumors to have low and intermediate grade tumors included under the term neuroendocrine neoplasm, and high grade tumors designated neuroendocrine carcinoma [2]. Of all GI tract NETs, the most common are those of the small bowel (45%) [1]. GI NETs frequently metastasize into the liver, though NETs of primary hepatic origin are extremely rare [3].

Diagnosis of primary hepatic neuroendocrine tumors (PHNETs) requires histological confirmation of a NET as well as the exclusion of disease elsewhere, with final diagnosis often not achieved until after the tumor is resected. Ultrasound, CT, and MRI are typically all employed for characterization of these lesions. However, their appearance on diagnostic imaging can be highly variable, often mimicking more common hepatic malignancies such as cholangiocarcinoma, hepatocellular carcinoma, or hepatic metastases [4].

Here, we report a hepatic lesion presenting at our institution diagnosed as a PHNET. Since these tumors are exceedingly rare, a PHNET was not considered initially but was diagnosed after treatment and histological analysis. A previous case of PHNET was confirmed at our institution and presented with similar ambiguity on initial diagnostic workup. It has been reported previously by Gurung et al. [5] and is used here for comparison with the current case.

## 2. Case

A 31-year-old female presented with a two-year history of intermittent sharp epigastric pain that began to increase in frequency in recent months. Episodes of pain lasted for up to 24 hours and were associated with severe nausea and vomiting. She had recently returned from Africa where she had been working for the past three years and was treated for malaria on three separate occasions. Her past medical history was otherwise unremarkable and her family history was significant only for celiac disease. Laboratory studies demonstrated a lipase of 57 U/L (normal 16–65), an ALP of 196 U/L (normal 30–135), and a GGT of 178 U/L (normal 15–80), with otherwise normal liver enzymes. Workup for malaria, Hepatitis B and C, and celiac disease were all negative. An esophagogastroduodenoscopy was also performed and revealed no findings to explain the patient's abdominal pain.

**Figure 1:**
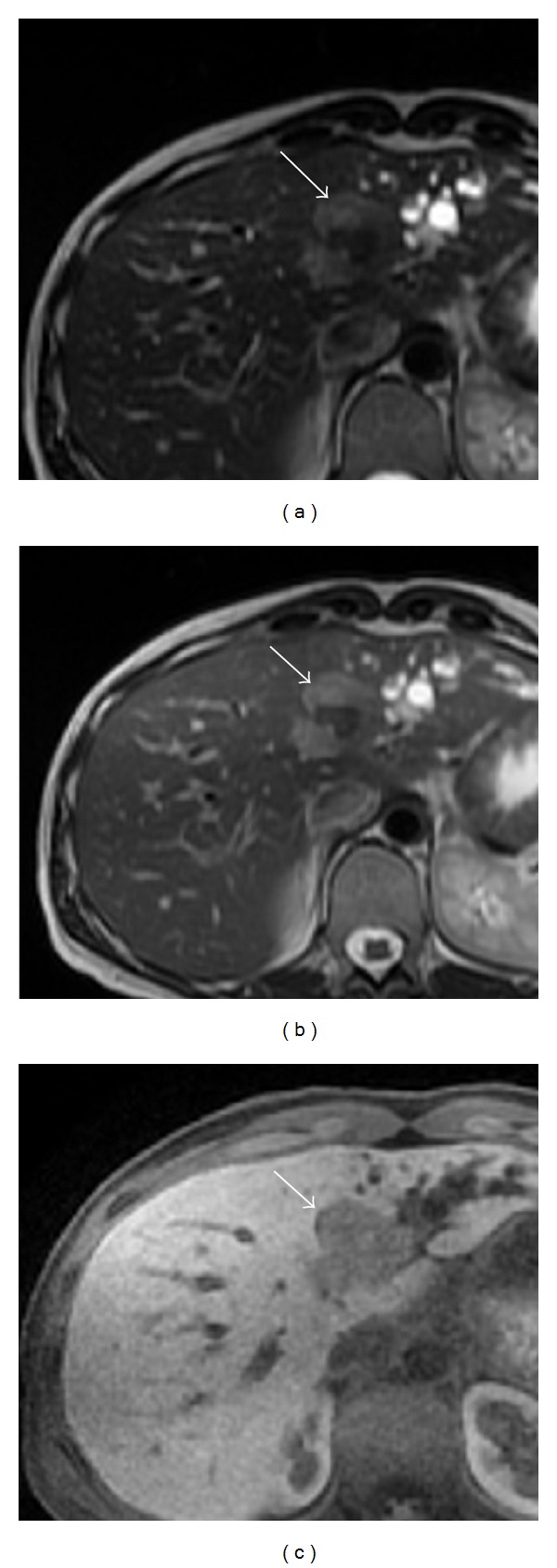
MRI of a lobulated lesion in segments 2 and 4 (white arrow). (a) T2 weighted first echo. (b) T2 weighted second echo. (c) Delayed liver acquisition with volume acquisition (LAVA).

An abdominal ultrasound was performed and demonstrated a 2 cm ill-defined echogenic lesion in the medial left lobe of the liver as well as marked intrahepatic bile duct dilatation. Subsequent MRI revealed a lobulated lesion involving segments 2 and 4 with mild hypervascularity on arterial phase images and washout on delayed images (Figure 1). There was effacement of the middle and left hepatic veins with associated duct dilatation.

Endoscopic retrograde cholangiopancreatography (ERCP) was performed to assess the patency of the biliary tree and revealed a focal left hepatic duct stricture with moderate to severe peripheral left biliary duct dilatation. Stenting of the stricture was performed and required later revision because of ongoing cholangitis.

**Figure 2:**
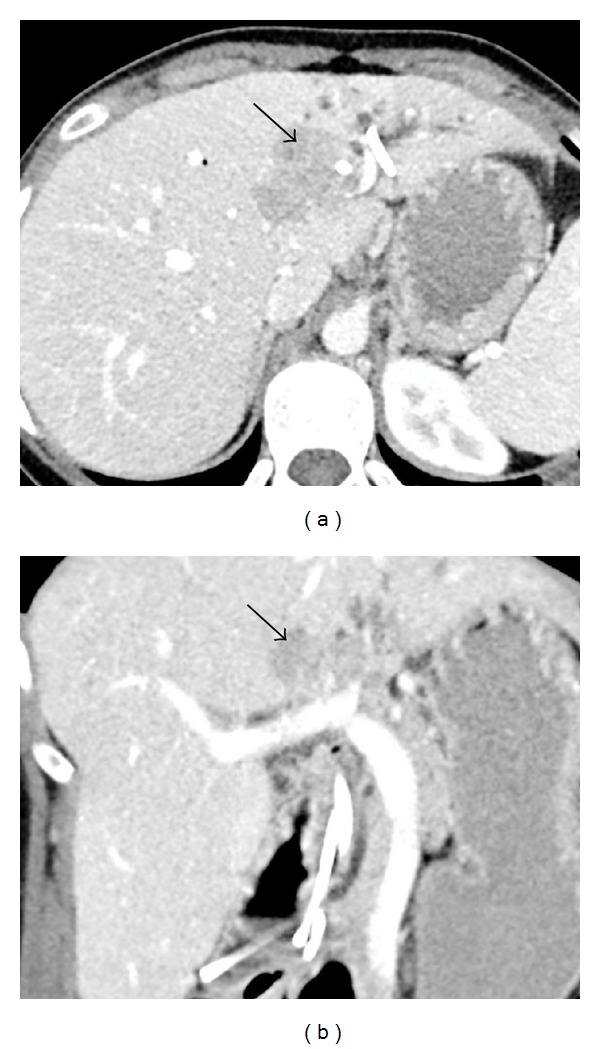
CT of the liver lesion (black arrow) in axial (a) and coronal (b) sections demonstrating an ill-defined heterogeneous soft tissue density in segment 4 as well as extensive intrahepatic biliary duct dilatation in segments 2 and 3 with pneumobilia.

CT was performed for tumor characterization, demonstrating an ill-defined heterogeneous soft tissue density mass in segment 4 of the liver measuring 4.2 × 3.8 cm as well as extensive intrahepatic biliary duct dilatation in segments 2 and 3 with pneumobilia (Figure 2). There was no evidence of disease elsewhere and no lymphadenopathy.

Based on the imaging, the presumed diagnosis was cholangiocarcinoma. The differential diagnoses for this lesion included hepatocellular carcinoma (HCC) and metastatic disease, though there was no obvious primary source.

Left hepatectomy with bile duct resection was performed, along with hepaticojejunostomy using the remaining right hepatic duct. No peritoneal carcinomatosis was noted upon exploration.

Grossly, the tumor was white-gray in appearance and more-or-less well circumscribed. It measured 4.5 × 3.5 × 4.5 cm. Immunohistochemistry was diffusely positive for synaptophysin and MIB-1 was positive in 5–10% of tumor cells. The tumor was determined to be an intermediate grade NET with a small amount of admixed adenocarcinoma in situ present within the bile duct. There was also evidence of nodal disease in two hepatic artery lymph nodes.

Postoperatively, the patient developed a collection of bilious fluid in the resection bed of her left hepatic lobe requiring the placement of a drain but subsequently did well following her surgery.

## 3. Discussion

Neuroendocrine neoplasms are a relatively rare tumor of the gastrointestinal system, accounting for less than 2% of all GI neoplasms [6]. PHNETs are exceedingly rare, with less than 300 cases reported in the literature to date [3]. WHO classification of tumors of the digestive system categorizes neuroendocrine neoplasms into three groups based on grade. Grades 1 and 2 represent low and intermediate grades, respectively, while high grade neuroendocrine neoplasms are termed neuroendocrine carcinomas [2]. TNM staging has been developed for neuroendocrine tumors and currently includes a staging system for gastric, small bowel, colonic, rectal, and ampulla of Vater neuroendocrine tumors; however, hepatic neuroendocrine tumors have yet to be included [7].

While the origin of PHNETs remains unclear, three hypotheses have been proposed: (1) they arise from neuroendocrine cells scattered in the epithelium of the intrahepatic biliary tract; (2) they originate from heterotopic pancreatic or adrenal tissue located in the liver; and (3) they arise from the neuroendocrine differentiation of a single malignant stem cell that is the precursor of other hepatic tumors [6].

Clinically, PHNETs have a clinical presentation that is distinct from other NETs. They are more frequent in women and more likely to affect the middle-aged population [6]. PHNETs appear most often as an endocrinologically silent hepatic mass or masses. This can be contrasted with hepatic metastases from extrahepatic NETs that are more commonly associated with typical carcinoid syndrome. The syndrome itself occurs as a result of the systemic release of neurosecretory products including primarily serotonin, but also histamine, bradykinin, and prostaglandins, and is characterized by flushing and diarrhea [6]. It remains unknown why primary NETs of the liver frequently remain endocrinologically silent while their metastatic counterparts do not. PHNETs are more often discovered based on symptoms related to mass effect on the liver and adjacent organs such as pain, weight loss, and palpable mass [1]. Our case presented with symptoms related to mass effect rather than carcinoid syndrome.

It is very difficult to differentiate PHNETs from other solid hepatic tumors, chiefly hepatocellular carcinoma (HCC) and cholangiocarcinoma, making postoperative histological analysis the main method for final diagnosis. Radiological imaging currently lacks specificity for these tumors, which are often mistaken for more common lesions. Ultrasound, CT, and MRI are all employed for lesion characterization; however, no formal techniques exist that are specific for PHNETs. Gross radiographic features can be highly variable with some lesions appearing solid or cystic, as well as having well-circumscribed or diffuse margins. Areas of necrosis have also been described [8]. Because of this highly variable appearance, a PHNET may be initially thought to be a benign lesion such as a hepatic adenoma or hemangioma or be confused with another hepatic malignancy such as HCC or cholangiocarcinoma. A recent case report by Krohn et al. even reports a PHNET mimicking an Echinococcus cyst on CT and MRI [9]. In an analysis of 11 cases, Huang et al. found that many of these tumors had cystic changes present on ultrasound, CT, and MRI. They suggested this may be helpful in differentiating them from HCC, which more commonly presents with liquefactive necrosis [10]. The case presented here did not demonstrate cystic changes. Interestingly, the lesion reported by Gurung et al. [5] did show a large area of central necrosis.

Limited CT and MRI characterization of PHNETs has revealed enhancement patterns similar to hepatic NET metastases from the pancreas or gastrointestinal tract [8]. On MRI, both primary tumors and metastases have been reported to appear hypointense on T1 weighted spin-echo sequences and hyperintense on T2 weighted fast spin-echo sequences [8]. Hepatic NET metastases also commonly show intense enhancement in hepatic arterial dominant phase with washout in portal venous and extracellular phases, reflecting hypervascularity [8]. Similar early enhancement has been shown in PHNETs. The MRI findings in our case are consistent with those described in the literature, though our lesion demonstrated only mild enhancement on hepatic arterial dominant phase. These patterns of MRI enhancement, however, are not specific to NETs and while they are helpful in characterization of the lesions, they do not differentiate them from HCC or cholangiocarcinoma.

Octreotide scanning has been shown to be a useful technique for identifying NETs, with a sensitivity of up to 90% and specificity near 83% [11, 12]. In addition, Gallium-68 somatostatin receptor positron emission tomography (PET) has recently been employed in the diagnosis of NETs. A meta-analysis (10 studies, 416 patients) performed by Yang et al. suggests high sensitivity and specificity of imaging agents 68Ga-DOTATOC (Sn 93%, Sp 85%) and 68Ga-DOTATATE (Sn 96%, Sp 100%) for diagnosing NETs on PET scan [13]. However, literature remains limited and further multicenter studies with larger sample sizes are needed to better evaluate the diagnostic performance of these two agents. Since patients with PHNETs are most likely to be devoid of the classic carcinoid syndrome, these diagnostic techniques may not be employed during initial workup.

Immunohistochemistry is performed as the definitive diagnosis for PHNETs, typically after they have already been resected. NETs have previously been shown to be associated with immunoreactivity for chromogranin A, neuron specific enolase, and synaptophysin [14]. The tumor in our case was immunoreactive for synaptophysin.

Treatment of PHNET has been largely reliant on surgery, with hepatectomy being the most effective treatment of localized tumors [12]. Transarterial chemoembolization (TACE) or liver transplantation has also been reported as effective options in those tumors that are confined to the liver but are unresectable [15–19]. TACE is an effective method for cytoreduction due to the high sensitivity of neuroendocrine tumors to ischemia [19]. Chemotherapy has also been proposed for treatment of PHNETs with multiple masses or distant metastases; however, its benefit remains questionable [18]. Given the size and location of the lesion in our case, hepatectomy was a feasible option for curative treatment. Since the extent of the other PHNET seen at our institution, reported by Gurung et al. [5], precluded curative hepatectomy, liver transplant was felt to be the best treatment option. Interestingly, pretransplant hepatic artery embolization did not demonstrate effective cytoreduction on follow-up CT in this case.

An overlap of imaging findings with other hepatic neoplasms means that diagnosis of PHNET continues to rest primarily on pathological analysis and immunohistochemistry following biopsy or resection. Given the typical lack of neuroendocrine symptoms as well as a nonspecific and variable radiographic appearance, PHNET will undoubtedly rarely be the favored diagnosis of a hepatic lesion discovered on imaging. However, it remains important to consider a NET when developing a differential diagnosis, especially if the lesion is not entirely typical of the other more common tumors.

Once a hepatic NET has been confirmed, a final diagnosis of PHNET must be that of exclusion. Only once a primary extrahepatic source is excluded can the diagnosis be made confidently. Surgery continues to be the treatment of choice for these rare tumors.
